# Academic AI overreliance scale: development and preliminary psychometric validation in health sciences university students

**DOI:** 10.3389/fdgth.2026.1882677

**Published:** 2026-07-21

**Authors:** Daniel Oleas, Jose A. Rodas, David Alarcón Rubio

**Affiliations:** 1 Department of Research, Universidad Ecotec, Samborondón, Ecuador; 2 Department of Social Anthropology, Basic Psychology and Public Health, Universidad Pablo de Olavide, Dos Hermanas, Sevilla, España; 3 School of Psychology, Universidad Espíritu Santo, Samborondón, Ecuador; 4 School of Psychology, University College Dublin, Dublin, Ireland

**Keywords:** academic autonomy, cognitive offloading, generative artificial intelligence, psychometric validation, scale development, self-regulated learning

## Abstract

**Introduction:**

The rapid integration of generative artificial intelligence (AI) into higher education has raised growing concerns regarding its potential impact on academic autonomy, self-regulated learning, and cognitive functioning. However, limited attention has been given to the progressive delegation of academic cognitive and self-regulatory processes to intelligent systems. This study aimed to develop and examine the initial psychometric properties of the Academic AI Overreliance Scale among health sciences university students.

**Methods:**

Two independent studies were conducted in Ecuador. In Study 1, an Exploratory Factor Analysis (EFA) was performed with 437 university students to identify the underlying structure of an initial 24-item pool. In Study 2, a Confirmatory Factor Analysis (CFA) was conducted with an independent sample of 437 university students to evaluate the factorial validity, measurement invariance, and internal consistency of the refined 22-item version.

**Results:**

Study 1 supported a two-factor solution composed of Emotional-Dysregulated Reliance and Cognitive Reliance. Study 2 confirmed the correlated two-factor model, showing excellent fit indices (CFI = 0.994, TLI = 0.994, RMSEA = 0.066, SRMR = 0.056) and strict invariance across sex groups. Internal consistency was adequate for both Emotional-Dysregulated Reliance (*α* = 0.94, *ω* = 0.94) and Cognitive Reliance (*α* = 0.88, *ω* = 0.88).

**Discussion:**

The findings suggest that Academic AI Overreliance may represent a specific form of cognitive and self-regulatory externalization associated with the integration of generative AI into academic activities, rather than a traditional form of technological addiction. The scale provides preliminary evidence of validity based on internal structure, measurement invariance, and reliability, and may serve as an initial instrument for future research on generative AI, academic reasoning, and intellectual autonomy in higher education.

## 1 Introduction

The integration of generative artificial intelligence (GAI) tools into higher education is profoundly transforming learning processes (1, 2), particularly in disciplines related to the health sciences (3). Unlike earlier forms of artificial intelligence primarily focused on classification or prediction, generative systems are capable of producing original natural-language content, including academic summaries, conceptual explanations, clinical analyses, and complex argumentative texts. These platforms can synthesize evidence, structure reasoning, and solve demanding tasks in real time, thereby altering the ways students access, organize, and process academic information (4, 5).

This phenomenon has generated growing interest within the field of medical and health sciences education, where debates are beginning to emerge regarding the potential benefits and risks of these technologies for professional training (6). In health sciences, the integration of generative AI is especially relevant because academic training depends not only on the acquisition of knowledge, but also on the progressive development of clinical reasoning, professional judgment, and decision-making under conditions of uncertainty (7). Current discussions surrounding the use of generative AI in education appear increasingly polarized, resembling the positions described by Umberto Eco in his debate between the “apocalyptic” and the “integrated” perspectives on mass culture (8). From an “integrated” perspective, AI is portrayed as a tool capable of democratizing access to knowledge, optimizing personalized learning, and improving academic efficiency (9, 10). Within this framework, AI functions as an advanced cognitive support system that amplifies students’ intellectual capacities and facilitates adaptive learning processes (11). In contrast, from a more “apocalyptic” standpoint, some scholars warn that intensive AI use may erode fundamental cognitive skills, diminish critical thinking, and promote forms of intellectual automation incompatible with deep learning (12, 13). These concerns are particularly salient in health sciences programs, where clinical reasoning fundamentally relies on the ability to integrate evidence, evaluate ambiguous information, and sustain complex decision-making processes.

Within this context, particularly in the fields of psychometrics and digital behavior assessment, attempts have begun to emerge to operationalize forms of intensive or dysfunctional AI use. Concepts such as “AI addiction” (14), “problematic AI use” (15), and “AI dependency” (16) have recently appeared in the literature as researchers attempt to adapt frameworks derived from problematic technology use and behavioral addictions to interactions with generative AI systems (17). However, traditional models of problematic technology use are generally grounded in mechanisms associated with hedonic gratification, reward-seeking, behavioral compulsion, and variable reinforcement (18, 19). Platforms such as social media and online video games primarily operate through dynamics designed to maximize emotional engagement and prolonged behavioral involvement (20). By contrast, generative AI in academic contexts appears to follow a different functional logic. Students do not use these tools exclusively for entertainment or emotional regulation, but also to delegate complex cognitive tasks directly linked to learning and academic performance (21).

This is particularly important because generative AI systems assume functions traditionally associated with advanced academic reasoning, including complex problem solving (22). These technologies increasingly function as forms of external cognitive support capable of progressively modifying how students develop intellectual competencies essential for their future professional practice (23). From this perspective, interpreting the phenomenon exclusively through addiction-based models may lead to conceptual overpathologization of AI use. Several authors have noted an ongoing debate regarding the contemporary tendency to pathologize everyday behaviors without sufficient evidence that they share the same psychopathological structure as classical addictions (24, 25). Within the context of AI, the central concern should lie in the progressive externalization of critical functions (13). This process may lead to a functional substitution of cognitive, self-regulatory, and academic processes that are essential for intellectual autonomy.

Academic AI Overreliance refers to a progressive functional displacement of academic cognitive and self-regulatory processes toward external intelligent systems. Accordingly, the present study conceptualizes Academic AI Overreliance as a persistent tendency to delegate intellectual and self-regulatory learning functions to generative AI systems in ways that may reduce autonomous cognitive engagement. This perspective seeks to move away from strictly pathologizing approaches and instead aligns with contemporary frameworks related to cognitive offloading, self-regulated learning, and trust in automation (26–28). Academic AI Overreliance should not be understood as frequent AI use *per se*, since recurrent use of generative AI may also reflect strategic and adaptive learning practices. The construct refers more specifically to a pattern in which AI assistance becomes linked to the delegation of academic reasoning and self-regulatory processes, particularly when students report difficulty sustaining learning tasks without such support. Accordingly, high scores should be interpreted as indicators of potential cognitive and self-regulatory delegation, not as evidence of cognitive impairment or clinically meaningful dysfunction.

From the perspective of Self-Regulated Learning theory, effective learning requires active processes of planning, monitoring, strategic control, and autonomous evaluation of academic performance (27). Academic AI Overreliance is therefore not equivalent to a general deficit in self-regulated learning, but rather describes a specific pattern in which these regulatory processes are increasingly delegated to generative AI during academic work. In this sense, students may come to rely on AI not only to obtain information, but also to initiate tasks, organize ideas, monitor progress, structure arguments, or evaluate the adequacy of their own academic output. In this regard, self-regulated learning deficits can occur independently of AI use, whereas Academic AI Overreliance refers specifically to the displacement of self-regulatory academic functions through generative AI-mediated assistance.

Academic AI Overreliance is also related to cognitive offloading. Cognitive offloading, understood as the use of external tools to reduce internal cognitive demands and optimize performance, traditionally in relation to devices such as calendars, calculators, search engines, or GPS systems (26). However, generative AI introduces a qualitative shift because it does not merely store, retrieve, or display information. Rather, it can interpret prompts, synthesize evidence, organize arguments, produce explanations, and generate complex academic text. Therefore, Academic AI Overreliance can be understood as a specific educational manifestation of cognitive offloading in which the externalized processes involve not only memory or information retrieval, but also higher-order academic operations such as reasoning, synthesis, conceptual organization, and problem solving (29).

Academic AI Overreliance also differs from academic procrastination and automation complacency, although it may intersect with both. Academic procrastination primarily refers to the unnecessary delay of academic tasks despite expected negative consequences (30, 31), whereas Academic AI Overreliance concerns how cognitive and self-regulatory processes are performed once students engage with academic work. A student may use AI because of procrastination, but overreliance refers to the functional substitution of academic reasoning rather than to delay itself. Similarly, the literature on Trust in Automation suggests that excessive reliance on automated systems may reduce critical monitoring, increase technological complacency, and diminish autonomy in decision-making processes (28). Academic AI Overreliance shares this concern, but it is situated specifically within generative AI-mediated learning, where excessive trust may weaken students’ active evaluation of AI-generated academic content.

Finally, By contrast, Academic AI Overreliance is defined more narrowly as an educational-psychological pattern involving the excessive delegation of academic cognitive and self-regulatory functions to generative AI systems. Thus, the construct is a theoretically specific form of cognitive and self-regulatory externalization that may affect academic autonomy, intellectual engagement, and professional training. This distinction is especially relevant in health sciences, where learning requires not only access to information, but also the progressive development of clinical reasoning, evidence integration, and decision-making under uncertainty.

### 1.1 The present study

Based on these theoretical foundations, the present study initially developed a multidimensional conceptualization of Academic AI Overreliance composed of four theoretical domains: cognitive externalization, self-regulatory dependence, emotional-functional dependence, and academic-social interference. This framework does not assume the existence of a clinical or subclinical addiction, but rather the possibility that the intensive integration of generative AI may progressively modify multiple dimensions of what is currently understood as academic autonomy among health sciences students. The cognitive externalization domain included indicators related to the progressive delegation of reasoning processes and reliance on automated synthesis. Self-regulatory dependence referred to difficulties initiating, sustaining, or controlling learning processes without continuous support from generative tools. The emotional-functional dimension sought to capture negative emotional responses associated with AI unavailability, whereas academic-social interference focused on potential functional consequences derived from the excessive use of these technologies within university life.

The aim of the present study was to develop and examine preliminary psychometric properties of a scale measuring Academic AI Overreliance among university students in the health sciences, evaluating the dimensional structure and internal consistency of the proposed construct.

## 2 Methodology

Two studies were conducted to develop and validate a scale designed to assess academic overreliance on artificial intelligence among university students. In the first study, an Exploratory Factor Analysis (EFA) was conducted to identify the underlying structure of the scale and refine the initial item pool. The preliminary version of the instrument was developed through expert review based on a theoretical literature review. This initial version was administered to a sample of health sciences university students. The EFA results indicated that the items tended to cluster into two primary dimensions.

In the second study, a Confirmatory Factor Analysis (CFA) was performed to examine the instrument's validity, structural invariance, and internal consistency. The full wording of the retained items and their factor allocation are provided in Supplementary Table S3. The CFA was conducted using an independent sample of university students and allowed for the evaluation of the stability and fit of the two-factor model identified in the EFA. To ensure transparency and reproducibility, the study methodology, database, analysis syntax, and full version of the scale were preregistered on Open Science Framework (OSF) https://osf.io/c9daw/.

### 2.1 Study 1

#### 2.1.1 Participants

The first sample consisted of 437 health sciences university students from a private university in Ecuador, ranging in age from 18 to 46 years (M = 21.65, SD = 3.78). Most participants were women (67.51%, *n* = 295), whereas 32.49% (*n* = 142) were men.

Participants were primarily enrolled in Nursing (38.22%), Health sciences programs (physical medicine and rehabilitation, nutrition, and radiology 24.94%), Medicine (18.76%), and Psychology (18.08%). More than half of the participants reported being unemployed (54.46%). The sample included students from the first through ninth semesters, with greater representation from the second (22.43%) and fourth semesters (17.16%). Regarding marital status, the vast majority identified as single (93.82%).

#### 2.1.2 Procedure and instruments

The initial construction of the scale was grounded in a theoretical integration of different approaches related to the use of digital technologies, as well as recent research examining the impact of generative artificial intelligence on educational and cognitive processes (6, 32). Based on this theoretical review, several conceptual domains were initially proposed to assess forms of academic overreliance on AI, including cognitive externalization, self-regulatory overreliance, emotional-functional dependence, and academic-social interference. Drawing on these domains, an initial pool of 32 items was developed using first-person statements specifically contextualized within university academic experiences, following classical recommendations for psychometric instrument development (33). The initial item pool and its theoretical organization are presented in Supplementary Table S1.

The wording of the items was informed by previous instruments related to artificial intelligence use, while prioritizing language that was clear and cultural adequacy for university students. Particular care was taken to avoid excessively technical expressions and the pathologization of artificial intelligence use. Subsequently, the initial item pool was evaluated by three expert judges with backgrounds in psychometrics, psychology, and digital education, following methodological recommendations for content validation procedures (34). The judges assessed the semantic clarity, conceptual relevance, theoretical coherence, and cultural appropriateness of each item. The review was qualitative in nature. Divergent observations were resolved through discussion within the research team, prioritizing conceptual specificity, non-pathologizing wording, and clarity for the target population. Based on their observations and recommendations, wording adjustments were made and eight items were removed due to conceptual redundancy or clarity issues, resulting in a preliminary 24-item version for empirical evaluation through exploratory factor analysis. Item order was randomized to reduce potential response biases associated with thematic clustering. The randomized item order used during administration are provided in the Supplementary Table S2.

Responses were recorded using a four-point Likert-type scale ranging from 1 (“Never happens to me”) to 4 (“Always happens to me”), with higher scores reflecting greater levels of academic overreliance on artificial intelligence. Four-point Likert scales have been recommended in psychological research because they balance response sensitivity while reducing central tendency bias (35).

The preliminary version of the scale, which also included informed consent and sociodemographic questions, was administered between September and October 2025 through an electronic questionnaire developed in Google Forms and completed in person in university classrooms. Before participating, all students provided electronic informed consent and were informed about the study objectives, the voluntary nature of participation, and the guarantees of confidentiality and anonymity. Data were collected anonymously and analyzed in R Studio (36). Descriptive analyses and the Exploratory Factor Analysis (EFA) were conducted primarily using the psych package (37).

#### 2.1.3 Data analysis

Initially, a preliminary item analysis was conducted using the first sample to examine response distributions and assess the initial psychometric adequacy of the item pool. Descriptive statistics were calculated for each item, including mean (M), standard deviation (SD), skewness, and kurtosis. Univariate normality was evaluated through skewness and kurtosis indices, with values within ±1.5 considered indicative of acceptable distributions for exploratory factor analyses (38).

Data suitability for factor analysis was subsequently assessed using the Kaiser-Meyer-Olkin (KMO) index and Bartlett's test of sphericity. Given the ordinal nature of the Likert-type items, a polychoric correlation matrix was estimated (39). The Exploratory Factor Analysis (EFA) was conducted on the polychoric correlation matrix using the minimum residuals (minres) extraction method with oblimin oblique rotation, given the expected correlation and residual correlation among the latent dimensions of the construct (40). The number of factors to retain was determined based on parallel analysis, and the theoretical interpretability of the factor solutions. During the factor refinement process, factor loadings, cross-loadings, and the conceptual coherence of the items were carefully examined. Items showing inadequate psychometric performance or theoretical inconsistencies were progressively removed.

The fit of the explored factor solutions was evaluated using multiple goodness-of-fit indices, including the chi-square statistic (*χ*^2^), Tucker–Lewis Index (TLI), Root Mean Square Error of Approximation (RMSEA), and Root Mean Square of Residuals (RMSR). Consistent with previous methodological recommendations, TLI values close to or greater than 90 were considered indicative of acceptable fit, whereas lower RMSEA and RMSR values reflected better model fit to the data (41). The sample size used in the present study was considered adequate for conducting robust factor analyses, as it substantially exceeded the commonly recommended minimum ratio 10 participants per item (42).

### 2.2 Study 2

#### 2.2.1 Participants

The second sample consisted of 437 health sciences university students from a private university in Ecuador. Participants ranged in age from 18 to 39 years (M = 21.30, SD = 2.98). Regarding sex, 69.34% (*n* = 303) were women and 30.66% (*n* = 134) were men. In terms of academic major, 37.76% (*n* = 165) of participants were enrolled in Nursing, 25.17% (*n* = 110) in Health sciences programs (physical medicine and rehabilitation, nutrition, and radiology) 19.22% (*n* = 84) in Medicine, and 17.85% (*n* = 78) in Psychology.

Regarding employment status, most participants reported not having a job (55.15%, *n* = 241), whereas the remaining students indicated different forms of employment or entrepreneurial activities. Participants represented different stages of academic training, ranging from the first to the ninth semester, with fourth-semester students constituting the largest subgroup (21.28%, *n* = 93). Finally, most participants identified as single (95.88%, *n* = 419).

#### 2.2.2 Procedure and instruments

In the second study, a refined 22-item version of the scale was used, organized into two dimensions: Cognitive Reliance and Emotional-Dysregulated Reliance, based on the results of the EFA conducted with the first sample. Participants responded using a four-point Likert-type scale ranging from 1 (“Never happens to me”) to 4 (“Always happens to me”). Participants were instructed to respond to all items based on their experiences during the current academic semester.

Data collection was administered between November and December 2025 at the same private university in Ecuador. The scale, which also included sociodemographic questions and informed consent, was administered through an electronic questionnaire developed in Google Forms and completed in person in university classrooms. All participants provided electronic informed consent prior to participation, and confidentiality, anonymity, and the exclusive academic use of the data were guaranteed throughout the study.

To evaluate the factorial structure of the instrument, a Confirmatory Factor Analysis (CFA) was conducted in RStudio using the lavaan (43) and semTools (44) packages. Factorial invariance across sex was also examined through progressively restrictive configural, metric, and scalar invariance models. Internal consistency was assessed using Cronbach's alpha and McDonald's omega coefficients, both considered appropriate reliability indicators for contemporary multidimensional instruments. Other potential grouping variables, such as academic program, semester, or frequency of AI use, were not tested because some categories were small or unevenly distributed, which could compromise parameter stability and the interpretation of invariance results.

#### 2.2.3 Data analysis

Descriptive statistics were initially calculated for all items. A Confirmatory Factor Analysis (CFA) was then performed using the Diagonally Weighted Least Squares (DWLS) estimator due to the ordinal nature of the Likert-type responses. This estimator is recommended for ordinal data because it relies on polychoric correlations and reduces the bias associated with Maximum Likelihood estimation under non-normal distributions (45). Model fit was evaluated using multiple fit indices, following the same criteria applied during the exploratory phase, and standardized factor loadings (*λ*) above.40 were considered acceptable.

Several factorial structures were tested to evaluate the dimensionality of the refined 22-item scale and to address the high association between the two latent dimensions. Specifically, we estimated a unidimensional model, a correlated two-factor CFA model, a bifactor model with a general Academic AI Overreliance factor and two orthogonal specific factors, and a two-factor exploratory structural equation model (ESEM). The unidimensional model was used to examine whether the item set could be adequately represented by a single general factor. The bifactor model was estimated to evaluate whether a general factor accounted for most of the common variance while retaining meaningful specific factors. The ESEM model was estimated to assess whether the high CFA factor correlation could be partly attributable to the restrictive assumption that all cross-loadings were fixed to zero. All models were estimated using the DWLS/WLSMV approach for ordered-categorical indicators. Model comparison was based on both statistical and theoretical criteria, including global fit indices, standardized factor loadings, factor correlations, specific-factor variance, cross-loading patterns, parsimony, and substantive interpretability. After identifying the best-fitting model, measurement invariance analyses were conducted to examine the stability of the factorial structure across groups through progressively restrictive configural, metric, and scalar models. Invariance was considered supported when changes in fit indices were within recommended thresholds (*Δ*CFI ≥ −.01 and *Δ*RMSEA ≤ 0.015) and chi-square difference tests were non-significant (46). Internal consistency was assessed using Cronbach’s alpha (*α*) and McDonald’s omega (*ω*), with coefficients above.70 considered indicative of adequate reliability (47, 48). To further examine convergent and discriminant validity, average variance extracted (AVE), composite reliability (CR), the Fornell-Larcker criterion, and the heterotrait-monotrait ratio of correlations (HTMT) were calculated for the correlated two-factor CFA model. AVE values above .50 were considered evidence of convergent validity, whereas discriminant validity was evaluated by comparing the square root of AVE with the latent interfactor correlation and by inspecting whether the HTMT value was below the conservative threshold of .85.

## 3 Results

### 3.1 Study 1

#### 3.1.1 Item reduction

Following expert review, eight items were removed due to redundancy and clarity concerns, resulting in a preliminary 24-item pool for exploratory factor analysis. Additional item refinement was conducted during the EFA based on psychometric performance and theoretical interpretability.

#### 3.1.2 Descriptive analysis of the items and exploratory factor analysis

Table 1 presents the descriptive statistics and exploratory factor analysis results for the preliminary 24-item pool. The polychoric correlation matrix showed excellent factorability, supported by a Kaiser–Meyer–Olkin index of .95 and a significant Bartlett's test of sphericity, *χ*^2^(276) = 8256.46, *p* < 0.001.

**Table 1:** Descriptive statistics and exploratory factor analysis results for the preliminary 24-item pool.

Item	M(SD)	g1	g2	F1	F2
1	2.44 (0.83)	0.08	−0.56		.69
2	2.39 (0.94)	0.17	−0.87		.70
3	2.18 (0.88)	0.32	−0.63		.67
4	2.25 (0.88)	0.23	−0.68		.68
5	2.18 (0.98)	0.31	−0.97		.42
6	1.75 (0.87)	0.97	0.14	.60	
7	2.22 (0.88)	0.29	−0.66		.71
8	1.91 (0.90)	0.69	−0.40	.46	
9	2.10 (0.84)	0.35	−0.54	.34	.47
10	2.06 (0.86)	0.39	−0.59	.34	.46
11	1.91 (0.87)	0.68	−0.33	.51	
12	1.78 (0.82)	0.72	−0.36	.76	
13	1.76 (0.87)	0.84	−0.28	.78	
14	1.74 (0.86)	0.90	−0.14	.78	
15	2.05 (0.88)	0.37	−0.75	.45	.43
16	1.81 (0.87)	0.78	−0.34	.72	
17	1.67 (0.83)	1.06	0.29	.92	
18	1.75 (0.83)	0.84	−0.13	.84	
19	1.67 (0.82)	1.01	0.22	.89	
20	1.88 (0.86)	0.66	−0.38	.60	
21	1.68 (0.83)	0.98	0.02	.91	
22	2.41 (0.97)	0.13	−0.98		.68
23	1.77 (0.86)	0.93	0.10	.73	
24	1.81 (0.82)	0.63	−0.50	.67	

g1, skewness; g2, kurtosis; F1, emotional-dysregulated reliance; F2, cognitive reliance. Only factor loadings (*λ*) greater than .30 are displayed.

An initial exploratory solution based on the theoretically proposed four-domain structure was examined. However, the four-factor solution showed several cross-loadings, substantial overlap between factors, and a weak fourth dimension. Parallel analysis subsequently supported a more parsimonious two-factor structure. The final exploratory factor analysis was estimated using the minimum residuals extraction method with oblimin rotation. The exploratory solution showed mixed fit evidence (RMSR = 0.03, TLI = 0.88, RMSEA = 0.088, BIC = −390.45). Although the RMSEA exceeded conventional criteria for close approximate fit, it was treated as one diagnostic indicator within a broader evaluation of the model rather than as an isolated rejection criterion. The solution was therefore retained because it was supported by adequate factorability, a theoretically coherent factor configuration, a clear pattern of item loadings, and subsequent cross-validation through CFA in an independent subsample. Conceptually, the first factor grouped items related to emotional-dysregulated reliance on AI, whereas the second factor reflected cognitive reliance and delegation of academic processes to AI systems.

### 3.2 Study 2

#### 3.2.1 Descriptive analysis of the items

Table 2 presents the descriptive statistics for the 22 items retained for confirmatory analyses. Item means ranged from 1.68 to 2.48, indicating moderate endorsement levels across the scale. Skewness and kurtosis values were generally within acceptable ranges, although several items showed moderate positive skewness, supporting the use of estimation methods appropriate for ordinal and non-normally distributed data.

**Table 2:** Descriptive statistics for the final 22-item scale.

Item	M (SD)	g1	g2
1	2.48 (0.88)	0.03	−0.71
2	2.41 (0.92)	0.11	−0.82
3	2.26 (0.91)	0.22	−0.79
4	2.30 (0.94)	0.25	−0.82
6	1.78 (0.90)	0.93	−0.05
7	2.21 (0.88)	0.30	−0.62
8	1.80 (0.87)	0.90	0.05
9	2.12 (0.87)	0.38	−0.58
10	2.15 (0.93)	0.37	−0.77
11	1.97 (0.88)	0.60	−0.41
12	1.78 (0.85)	0.90	0.08
13	1.78 (0.88)	0.85	−0.22
14	1.72 (0.85)	1.02	0.25
15	2.06 (0.89)	0.44	−0.62
16	1.86 (0.88)	0.75	−0.28
17	1.68 (0.87)	1.08	0.23
18	1.74 (0.84)	0.98	0.31
19	1.71 (0.80)	0.96	0.35
20	1.89 (0.87)	0.69	−0.31
22	2.37 (0.98)	0.21	−0.95
23	1.81 (0.88)	0.82	−0.19
24	1.88 (0.86)	0.73	−0.15

g1, skewness; g2, kurtosis.

#### 3.2.2 ESEM and confirmatory factor analysis

Confirmatory Factor Analyses were conducted using the DWLS/WLSMV estimator to evaluate the factorial validity of the refined 22-item scale. Given the high correlation observed between the two dimensions, we compared several alternative measurement structures: a unidimensional model, a correlated two-factor CFA model, a bifactor model, and a two-factor ESEM model. Table 3 presents the goodness-of-fit indices for all models. The unidimensional model showed the weakest fit among the tested solutions, suggesting that a strictly single-factor representation did not adequately reproduce the covariance structure of the items. The correlated two-factor CFA model showed improved fit relative to the unidimensional model and produced statistically significant and substantively meaningful standardized loadings across both dimensions. However, the two factors were strongly correlated, r = 0.87, indicating substantial overlap between Cognitive Reliance and Emotional-Dysregulated Reliance. The bifactor model showed the best global fit indices. Nevertheless, inspection of the parameter estimates indicated limited interpretability of the specific factors after accounting for the general Academic AI Overreliance factor. In particular, the Emotional-Dysregulated specific factor showed non-significant residual variance, and several item loadings on the specific factors were weak or non-significant. Therefore, despite its superior global fit, the bifactor model was not retained as the final measurement model.

**Table 3:** Goodness-of-Fit indices for the confirmatory factor models.

Model	*χ* ^2^	df	CFI	TLI	RMSEA	90% CI RMSEA	SRMR
Total sample – Unidimensional model	826.567	209	.991	.990	.082	[.076, .088]	.068
Total sample – Correlated two-factor model	603.01	208	.994	.994	.066	[.060, .072]	.056
Total sample – Bifactorial model	237.817	187	.999	.999	.025	[.014, .034]	.035
Total sample – ESEM model	236.159	188	.999	.999	.024	[.013, .033]	.035
Men – Correlated two-factor model	300.91	207	.995	.994	.058	[.043, .072]	.074
Women – Correlated two-factor model	505.62	207	.994	.994	.069	[.062, .077]	.059

CFI, comparative fit index; TLI, tucker–lewis index; RMSEA, root mean square error of approximation; SRMR, standardized root mean square residual. DWLS estimation was used for all models.

The two-factor ESEM model was estimated to examine whether the high CFA factor correlation could be partly explained by the restriction of all cross-loadings to zero. The ESEM model showed improved fit relative to the correlated two-factor CFA model and reduced the factor correlation from.87 to .64. This suggests that the high correlation observed in the CFA model was partly attributable to unmodeled cross-loadings rather than complete redundancy between the two dimensions. However, because several items showed meaningful secondary loadings, the ESEM solution was interpreted as a sensitivity analysis rather than as the final retained model. Overall, the correlated two-factor CFA model was retained as the final measurement structure because it provided better fit than the unidimensional model, avoided the limited interpretability observed in the bifactor solution, preserved a clearer and more parsimonious item-factor structure than the ESEM solution, and remained consistent with the theoretical distinction between cognitive delegation and emotional-dysregulated reliance on AI.

Although the two latent dimensions were strongly correlated in the CFA model [r = 0.87; 95% CI (.849, .894)], the additional model comparisons supported retaining the correlated two-factor structure. The unidimensional model showed poorer fit, indicating that the scale was not optimally represented by a single undifferentiated factor. The bifactor model suggested the presence of a strong general Academic AI Overreliance factor, but the weak interpretability of the specific factors limited its suitability as the final model. The ESEM model further showed that the factor correlation decreased when cross-loadings were freely estimated, suggesting that the high CFA correlation partly reflected restrictive zero cross-loading assumptions. Therefore, the two-factor CFA model was retained not because the dimensions were independent, but because it offered the best balance between empirical adequacy, parsimony, theoretical interpretability, and stability of parameter estimates.

#### 3.2.3 Convergent and discriminant validity

Additional analyses were conducted to evaluate convergent and discriminant validity for the retained correlated two-factor CFA model. Convergent validity was supported, as AVE values exceeded the recommended threshold of .50 for both Cognitive Reliance (AVE = 0.527) and Emotional-Dysregulated Reliance (AVE = 0.658). Composite reliability was also high for both dimensions, with CR = 0.908 for Cognitive Reliance and CR = 0.961 for Emotional-Dysregulated Reliance, indicating strong internal consistency of the latent constructs.

Regarding discriminant validity, the Fornell-Larcker criterion was not fully met. The square roots of AVE were .726 for Cognitive Reliance and .811 for Emotional-Dysregulated Reliance, both of which were lower than the latent interfactor correlation observed in the CFA model (r = 0.873). However, the HTMT ratio was .815, which is below the conservative threshold of .85, supporting discriminant validity according to this more specific criterion. Taken together, these findings indicate that Cognitive Reliance and Emotional-Dysregulated Reliance are strongly associated but not empirically redundant. Therefore, the two-factor solution was retained as a theoretically meaningful and psychometrically defensible representation of Academic AI Overreliance.

#### 3.2.4 Measurement invariance across Sex

**Figure 1:**
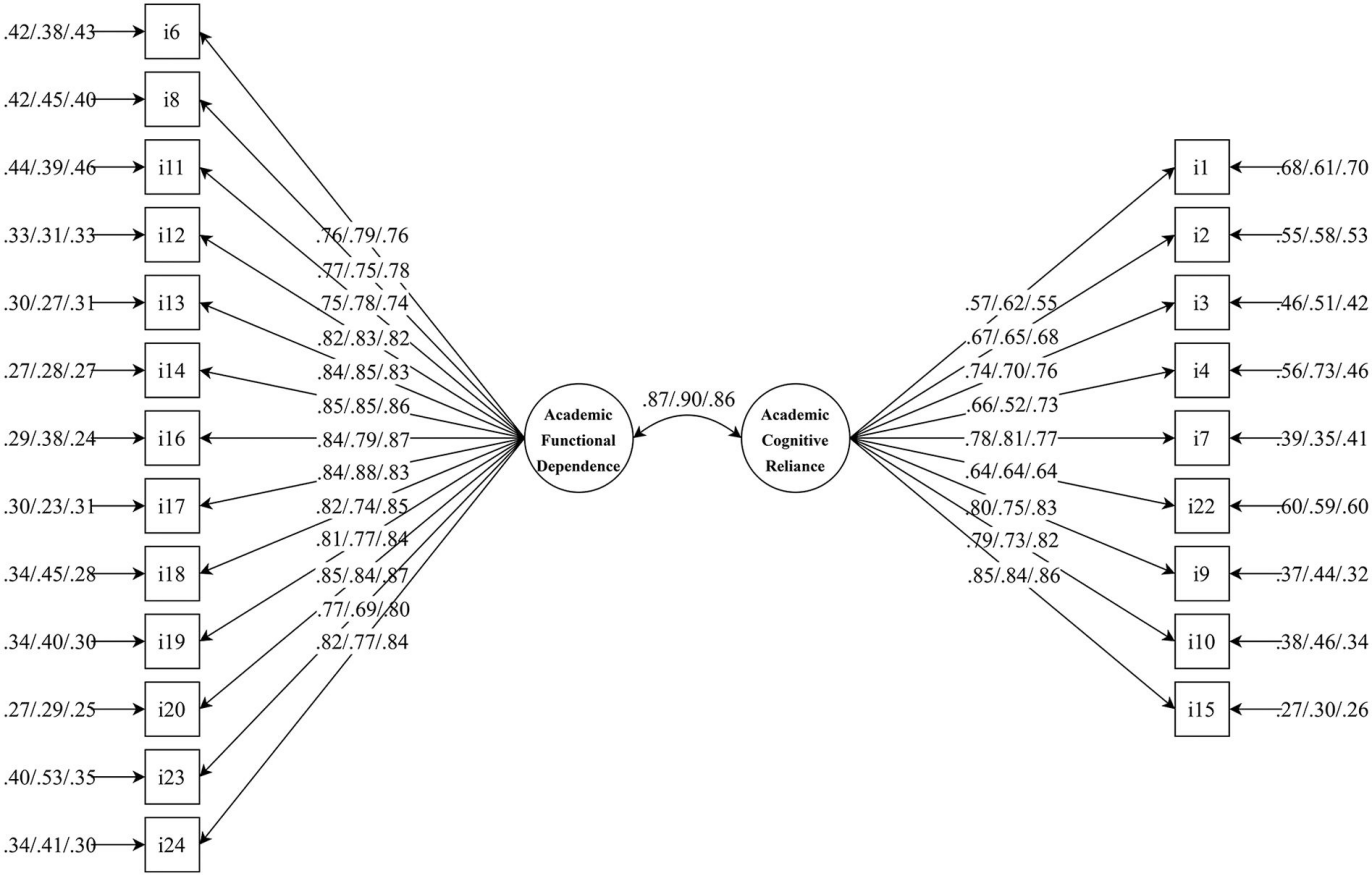
Standardized factor loadings of the academic overreliance on artificial intelligence scale: total sample (left), male participants (center), and female participants (right). Values separated by slashes correspond to the total sample, male participants, and female participants, respectively. Standardized factor loadings are displayed along the paths, whereas values adjacent to the observed variables represent residual variances. All standardized loadings were statistically significant (*p* < 0.001).

Measurement invariance across sex was evaluated through a sequence of increasingly constrained models, including configural, metric, scalar, and strict invariance. Following current recommendations for ordinal data, invariance was primarily assessed through changes in approximate fit indices between nested models. As presented in Table 4, the configural model demonstrated adequate fit, indicating that the factorial structure was similar across male and female participants. Subsequent metric, scalar, and strict invariance models also showed acceptable fit indices. Importantly, changes in CFI, RMSEA, and SRMR across nested models remained within recommended thresholds, supporting the equivalence of factor loadings, thresholds, and residuals across sex groups. Overall, these findings provide evidence for the structural stability of the scale and support the comparability of scores between male and female participants. The standardized factor loadings for the total sample and by sex are presented in Figure 1.

**Table 4:** Measurement invariance across Sex.

Model	CFI	TLI	RMSEA	SRMR	*Δ*CFI	*Δ*RMSEA	*Δ*SRMR
Configural	.995	.994	.066	.063	—	—	—
Metric	.993	.992	.075	.069	−0.002	.009	.005
Scalar	.995	.995	.062	.063	.002	−0.013	−0.005
Strict	.995	.995	.062	.063	.000	.000	.000

CFI, comparative fit index; TLI, tucker–lewis index; RMSEA, root mean square error of approximation; SRMR, standardized root mean square residual. *Δ* values represent changes relative to the previous nested model. Measurement invariance was evaluated following conventional recommendations for ordinal data. Changes in fit indices remained within recommended thresholds across all invariance levels, supporting configural, metric, scalar, and strict invariance across sex groups.

#### 3.2.5 Internal consistency

Internal consistency was evaluated using Cronbach's *α* and McDonald's *ω* coefficients. The Emotional-Dysregulated Reliance dimension showed *α* = 0.94 and *ω* = 0.94, whereas the Cognitive Reliance dimension presented *α* = 0.88 and *ω* = 0.88.

## 4 Discussion

The present study aimed to develop and examine initial psychometric evidence for a scale designed to assess Academic AI Overreliance among health sciences university students. Overall, the findings support the existence of a bidimensional structure composed of the dimensions Emotional-Dysregulated Reliance and Cognitive Reliance, both characterized by adequate fit indices, factorial validity, and invariance across men and women. Taken together, these findings suggest that the use of generative artificial intelligence systems in academic contexts is associated with specific transformations in how students organize, regulate, and delegate processes related to learning and academic reasoning. Beyond assessing frequent technology use, or even problematic use (including Frankenstein Syndrome (49);, the instrument captures particular forms of cognitive and self-regulatory externalization associated with the progressive integration of intelligent systems into everyday academic activities.

The first identified dimension, Emotional-Dysregulated Reliance, groups indicators associated with emotional discomfort, difficulties in academic self-regulation, and perceived functional interference when artificial intelligence is unavailable or cannot be used during study activities. Conceptually, this dimension relates more to alterations in self-regulated learning processes than to classical forms of technological dependence. Self-Regulated Learning theory proposes that effective learning requires active processes of planning, monitoring, and strategic control of one's own academic performance (27). From this perspective, the use of generative tools may progressively modify academic autonomy when certain functions related to idea organization, task initiation, or study regulation begin to shift toward external systems. In this sense, the obtained results reflect a possible vulnerability in the ability to sustain autonomous learning processes without constant support from intelligent tools. Nevertheless, the growing incorporation of these technologies into everyday life and their rapid normalization within educational settings also suggest the need to rethink how learning processes will be conceived in the future (50). In this new scenario, generative artificial intelligence functions not only as an academic support tool, but also as a system capable of transforming the distribution of functions between students and intelligent systems.

On the other hand, the Cognitive Reliance dimension probably constitutes one of the most conceptually relevant findings of the study. This factor groups items related to the delegation of academic reasoning processes, conceptual synthesis, and intellectual structuring toward generative artificial intelligence systems. In this sense, generative AI actively participates in higher-order cognitive operations, including reasoning, content organization, problem solving, and the production of complex texts (5, 22). These findings are consistent with the cognitive offloading framework, which suggests that individuals tend to transfer mental processes to external tools in order to reduce cognitive load and optimize performance (26). However, generative artificial intelligence introduces an important qualitative difference, as cognitive externalization is no longer limited to peripheral instrumental functions and instead begins to extend toward central processes of academic and professional reasoning. In this sense, the results appear to support the idea of a process of cognitive-functional displacement, understood as a progressive redistribution of cognitive and self-regulatory functions from the student toward external intelligent systems.

During the validation of the instrument, both dimensions showed a high correlation, indicating that they represent closely related components of the same broader phenomenon rather than independent constructs. However, the findings do not necessarily support a strictly unidimensional interpretation. This interpretation was further supported by the discriminant validity analyses. Although the Fornell-Larcker criterion was not satisfied, reflecting the substantial latent overlap between the two dimensions, the HTMT ratio remained below the conservative.85 threshold. Thus, the evidence suggests that the dimensions should not be interpreted as independent constructs, but rather as closely connected yet empirically distinguishable components of the broader Academic AI Overreliance phenomenon. Cognitive Reliance reflects the delegation of academic thinking processes to generative AI systems, including reasoning, synthesis, organization, and problem solving, whereas Emotional-Dysregulated Reliance captures the emotional discomfort, regulatory difficulty, and perceived academic interference associated with relying on such systems. The strong association between both dimensions is therefore expected, because repeated cognitive delegation may progressively become linked to emotional dependence and reduced confidence in autonomous academic performance. Nevertheless, the distinction remains relevant because the two dimensions point to different educational processes and intervention targets: one related to cognitive and self-regulatory delegation, and the other to emotional reliance and academic interference. This distinction is important because the emotional and functional components of the scale may partially resemble dependency-related constructs at the descriptive level.

Nevertheless, their theoretical meaning differs within the present framework. In addiction-based models, discomfort and interference are generally interpreted in relation to compulsive engagement, craving, withdrawal-like experiences, loss of control, or reinforcement processes (18, 19). In contrast, Academic AI Overreliance is organized around a different core mechanism: the functional displacement of academic cognitive and self-regulatory processes toward generative AI systems. This interpretation is consistent with the concept of Trust in Automation, which suggests that excessive reliance on automated systems may reduce monitoring, increase complacency, and weaken autonomous decision-making (28). Thus, although Academic AI Overreliance may overlap phenomenologically with problematic AI use or AI dependency, it remains theoretically and empirically distinct insofar as its retained items are specifically anchored in academic reasoning, learning regulation, and the delegation of study-related functions, rather than in generalized compulsive use, hedonic reinforcement, or addiction-like dependency.

The implications of these findings may be especially relevant within the health sciences. Training in disciplines such as medicine, nursing, or psychology depends not only on the acquisition of declarative knowledge, but also on the progressive development of clinical reasoning, professional judgment, and decision-making under conditions of uncertainty (7). Within these disciplines, the integration of Generative Artificial Intelligence may partially modify how students develop competencies related to critical thinking, evidence integration, and intellectual autonomy. In this sense, the developed instrument could constitute a useful tool for future research aimed at understanding how the progressive incorporation of generative artificial intelligence transforms university learning processes and the relationship between students and intelligent systems. Likewise, the fact that the scale demonstrated strict invariance across men and women strengthens the structural stability of the instrument and supports the comparability of scores across groups.

Finally, this study presents several limitations. First, both samples were composed exclusively of health sciences students from a private university in Ecuador, which limits the generalizability of the findings to other institutional, academic, and cultural contexts. Although the sample sizes were adequate for the exploratory and confirmatory analyses, the results should be interpreted as preliminary and context-specific. In addition, both samples showed a predominance of female participants, reflecting the composition of the participating programs but limiting the extent to which the findings can be generalized to more balanced sex distributions. Future studies should examine the scale in public universities, universities from different countries, non-health-related disciplines, postgraduate students, and samples with more balanced representation of men and women. Such studies would help determine whether the factorial structure, measurement invariance, and reliability of the scale remain stable across broader educational contexts. In addition, measurement invariance was examined only across sex. Although this provides initial evidence regarding the comparability of scores between male and female participants, future studies should evaluate invariance across other relevant groups, including academic program, academic semester, level of AI use, type of AI tool used, and educational context. This would help determine whether the factorial structure and item functioning of the scale remain stable across different academic trajectories and patterns of AI engagement. Future studies should also distinguish empirically between adaptive AI-assisted learning and maladaptive overreliance, as frequent use of generative AI may support learning when accompanied by active monitoring, critical evaluation, and autonomous engagement, whereas overreliance would require evidence of functional interference, reduced self-regulation, or poorer academic outcomes.

Second, the present study provides validity evidence primarily based on internal structure, internal consistency, and measurement invariance. Although these sources of evidence are essential during the initial development of a new instrument, they are not sufficient to establish the broader construct validity of the scale. Because no external measures were included, convergent, discriminant, criterion-related, and predictive validity could not be examined. This limitation is particularly relevant because Academic AI Overreliance is theoretically adjacent to constructs such as self-regulated learning, academic procrastination, cognitive offloading, AI literacy, academic engagement, critical thinking, academic performance, trust in automation, problematic technology use, technology dependence, and AI dependency. Future studies should therefore examine whether the scale shows theoretically expected associations with these constructs while also demonstrating empirical distinctiveness from broader dependence-related or problematic technology use measures. This is especially important to determine whether Academic AI Overreliance captures a specific pattern of academic cognitive and self-regulatory delegation to generative AI systems rather than merely reflecting generalized technology dependence or problematic AI use. Longitudinal designs are also needed to evaluate temporal stability, test–retest reliability, and the predictive value of Academic AI Overreliance for academic outcomes and changes in autonomous learning over time.

## 5 Conclusion

The development of the present scale represents an important step toward understanding how generative artificial intelligence is progressively integrated into academic functioning among university students. The instrument provides preliminary psychometric evidence based on internal structure, reliability, and structural stability across sex groups, supporting its potential usefulness for assessing Academic AI Overreliance in higher education contexts. However, these findings should be interpreted as an initial step in the validation process rather than as definitive evidence of the scale's broader validity. Beyond evaluating frequent or problematic technology use, the scale captures specific forms of cognitive and self-regulatory externalization associated with the incorporation of intelligent systems into everyday academic activities. In this sense, the findings contribute to the emerging literature examining how generative AI may transform learning processes, academic autonomy, and the distribution of cognitive functions between students and intelligent systems. Nevertheless, additional studies conducted in broader populations, different academic disciplines, and diverse cultural contexts are necessary to further examine the applicability and stability of the instrument. Expanding its use across different educational settings would not only strengthen the generalizability of the findings, but also contribute to a more comprehensive understanding of how generative artificial intelligence may influence academic reasoning, self-regulated learning, and intellectual autonomy in contemporary higher education. Such efforts may also help inform future educational strategies, institutional policies, and research initiatives aimed at promoting a more critical, autonomous, and balanced integration of artificial intelligence within university learning environments.

## Data Availability

The datasets presented in this study can be found in online repositories. The names of the repository/repositories and accession number(s) can be found in Open Science Framework https://osf.io/c9daw/.
