# Supplementary material for: Academic AI overreliance scale: development and preliminary psychometric validation in health sciences university students
Source: Front Digit Health. 2026 Jul 21;8:1882677. doi: 10.3389/fdgth.2026.1882677 (PMC13433740; doi:10.3389/fdgth.2026.1882677)
Supplement: Supplementary file 1 [file Datasheet1.pdf]

frontiers

Supplementary Material

Supplementary Table 1 Initial Item Pool and Theoretical Domains

Spanish	English	Factor
Intento limitar el tiempo que utilizo la IA en mis estudios, pero siempre termino usándola más de lo que planeé.	I try to limit the amount of time I use AI for my studies, but I usually end up using it more than I planned.	Self-Regulatory Overreliance
Siento que tengo que consultar a la IA inmediatamente al recibir una tarea académica, antes de intentar resolverla por mí mismo/a.	I feel the need to consult AI immediately when I receive an academic assignment, before trying to solve it on my own.	
He llegado a utilizar la IA en horarios inadecuados (como muy tarde en la noche o durante comidas) para completar pendientes académicos.	I have used AI at inappropriate times (e.g., late at night or during meals) to complete academic work.	
Me resulta difícil concentrarme en la lectura o el estudio si no tengo abierta una herramienta de IA lista para ser consultada.	I find it difficult to concentrate on reading or studying if I do not have an AI tool open and readily available.	
Cuando tengo una tarea difícil, mis pensamientos giran constantemente en torno a cómo voy a usar la IA para resolverla.	When I face a difficult academic task, my thoughts constantly revolve around how I will use AI to solve it.	
Antes de intentar resolver una actividad académica por mi cuenta, suelo abrir automáticamente una herramienta de IA para orientarme o empezar la tarea.	Before trying to solve an academic activity on my own, I tend to automatically open an AI tool for guidance or to get started.	
Aunque planeo usar la IA solo como apoyo puntual, termino dependiendo de ella durante gran parte de mi proceso de estudio o elaboración de trabajos.	Although I intend to use AI only as occasional support, I end up relying on it throughout much of my studying or assignment-writing process.	
El tiempo que dedico a interactuar con la IA para la escuela me ha hecho descuidar otras responsabilidades o actividades de estudio importantes.	The time I spend interacting with AI for schoolwork has caused me to neglect other responsibilities or important study activities.	
Me siento mentalmente agotado/a si tengo que elaborar un texto argumentativo largo sin la asistencia directa de la IA.	I feel mentally exhausted when I have to write a long argumentative text without direct AI assistance.	Emotional-Functional Overreliance

Si mi acceso a las herramientas de IA se interrumpe (e.g., por falta de internet), me siento inmediatamente ansioso/a o frustrado/a.	If my access to AI tools is interrupted (e.g., due to internet problems), I immediately feel anxious or frustrated.	
Me pongo irritable o de mal humor si un profesor me pide completar una tarea en la que está prohibido usar cualquier tipo de IA.	I become irritable or frustrated when a professor asks me to complete an assignment in which any type of AI use is prohibited.	
He sentido un "vacío" o "bloqueo" mental al intentar estudiar o escribir sin tener la IA como soporte inmediato.	I have experienced a sense of "mental emptiness" or "blockage" when trying to study or write without AI as immediate support.	
Para evitar la ansiedad, prefiero pagar o buscar una IA avanzada, aunque tenga que dedicarle un tiempo considerable a ello.	To avoid anxiety, I prefer to pay for or seek out more advanced AI tools, even if it requires considerable time or effort.	
Si la IA me da un error o una respuesta irrelevante, siento un alto nivel de frustración que me dificulta seguir estudiando.	If AI provides an error or an irrelevant response, I experience a high level of frustration that makes it difficult for me to continue studying.	
Cuando no tengo acceso inmediato a una herramienta de IA, siento inseguridad sobre mi capacidad para resolver adecuadamente las tareas académicas por mi cuenta.	When I do not have immediate access to an AI tool, I feel insecure about my ability to complete academic tasks adequately on my own.	
Siento que si se me exigiera completar una materia sin absolutamente ninguna ayuda de IA, mi rendimiento se vería seriamente afectado.	I feel that if I were required to complete a course without any AI assistance at all, my academic performance would be seriously affected.	
Dejo de lado métodos de estudio más tradicionales (libros, apuntes) porque la IA me ofrece una solución más rápida y prioritaria.	I set aside more traditional study methods (e.g., books or class notes) because AI provides a faster and more immediate solution.	Cognitive Externalization
Si se me ocurre una idea para un ensayo o proyecto, mi primer impulso es pedirle a la IA que la desarrolle o la estructure.	When I come up with an idea for an essay or project, my first impulse is to ask AI to develop or structure it.	
Cada vez necesito usar la IA para tareas académicas más complejas (análisis crítico, síntesis, argumentación) que antes podía hacer solo/a.	I increasingly rely on AI for more complex academic tasks (e.g., critical analysis, synthesis, or argumentation) that I used to handle on my own.	
Confío ciegamente en las respuestas y resúmenes de la IA, sin tomar el tiempo de verificar la información en fuentes primarias.	I trust AI-generated answers and summaries without taking the time to verify the information using primary sources.	

Las respuestas que genero sin ayuda de la IA me parecen insuficientes o de menor calidad que las que obtengo con su apoyo.	The responses I produce without AI assistance seem less adequate or lower in quality than those I generate with its support.	
Cuando leo textos, me cuesta trabajo identificar las ideas centrales sin que la IA me genere un resumen o "puntos clave".	When reading texts, I find it difficult to identify the main ideas unless AI provides me with a summary or “key points.”	
Si la IA me da un resultado, me cuesta mucho editarlo o reescribirlo, prefiero usarlo casi tal cual lo generó.	When AI generates a result for me, I find it difficult to substantially edit or rewrite it and prefer to use it almost exactly as generated.	
Sé que al usar la IA estoy limitando el desarrollo de mis propias habilidades cognitivas (pensamiento crítico, síntesis), pero sigo usándola.	I am aware that using AI may limit the development of my own cognitive skills (e.g., critical thinking or synthesis), yet I continue to rely on it.	
Debido a mi dependencia de la IA, a veces entrego trabajos que no reflejan mi nivel real de comprensión o conocimiento de la materia.	Due to my reliance on AI, I sometimes submit assignments that do not reflect my actual level of understanding or knowledge of the subject.	Academic-Social Interference
He tenido conflictos o he estado en riesgo de problemas éticos (plagio) debido a la manera en que he utilizado la IA en mis trabajos.	I have experienced conflicts or been at risk of ethical issues (e.g., plagiarism) because of the way I have used AI in my academic work.	
Mis interacciones con compañeros de clase sobre tareas han disminuido, pues prefiero obtener la solución directamente de la IA.	My interactions with classmates about academic tasks have decreased because I prefer obtaining solutions directly from AI.	
He participado menos activamente en clases o debates porque siento que puedo resolver las tareas más rápido usando IA por mi cuenta.	I have participated less actively in classes or discussions because I feel I can complete tasks more quickly using AI on my own.	
El uso frecuente de IA ha reducido el tiempo que dedico a analizar o reflexionar profundamente sobre los contenidos académicos.	Frequent AI use has reduced the amount of time I spend analyzing or deeply reflecting on academic content.	
A veces dependo tanto de la IA que postergo el desarrollo de habilidades que debería practicar por mí mismo/a.	At times, I rely on AI so much that I postpone developing skills that I should be practicing on my own.	
Prefiero consultar a la IA antes que discutir ideas o resolver dudas con compañeros o docentes.	I prefer consulting AI before discussing ideas or resolving doubts with classmates or instructors.	
He entregado tareas realizadas con apoyo de IA sin revisar completamente si reflejan realmente mi comprensión del tema.	I have submitted assignments completed with AI support without fully reviewing whether they truly reflect my understanding of the topic.	

Supplementary Table 2 Administered Preliminary 24-Item Pool

Item	Spanish	English
1	Intento limitar el tiempo que utilizo la IA en mis estudios, pero siempre termino usándola más de lo que planeé.	I try to limit the amount of time I use AI for my studies, but I usually end up using it more than I planned.
2	Dejo de lado métodos de estudio más tradicionales (libros, apuntes) porque la IA me ofrece una solución más rápida y prioritaria.	I set aside more traditional study methods (e.g., books or class notes) because AI provides a faster and more immediate solution.
3	Siento que tengo que consultar a la IA inmediatamente al recibir una tarea académica, antes de intentar resolverla por mí mismo/a.	I feel the need to consult AI immediately when I receive an academic assignment, before trying to solve it on my own.
4	Si se me ocurre una idea para un ensayo o proyecto, mi primer impulso es pedirle a la IA que la desarrolle o la estructure.	When I come up with an idea for an essay or project, my first impulse is to ask AI to develop or structure it.
5	He llegado a utilizar la IA en horarios inadecuados (como muy tarde en la noche o durante comidas) para completar pendientes académicos.	I have used AI at inappropriate times (e.g., late at night or during meals) to complete academic work.
6	Me resulta difícil concentrarme en la lectura o el estudio si no tengo abierta una herramienta de IA lista para ser consultada.	I find it difficult to concentrate on reading or studying if I do not have an AI tool open and readily available.
7	Cada vez necesito usar la IA para tareas académicas más complejas (análisis crítico, síntesis, argumentación) que antes podía hacer solo/a.	I increasingly rely on AI for more complex academic tasks (e.g., critical analysis, synthesis, or argumentation) that I used to handle on my own.
8	Confío ciegamente en las respuestas y resúmenes de la IA, sin tomar el tiempo de verificar la información en fuentes primarias.	I trust AI-generated answers and summaries without taking the time to verify the information using primary sources.
9	Me siento mentalmente agotado/a si tengo que elaborar un texto argumentativo largo sin la asistencia directa de la IA.	I feel mentally exhausted when I have to write a long argumentative text without direct AI assistance.
10	Las respuestas que genero sin ayuda de la IA me parecen insuficientes o de menor calidad que las que obtengo con su apoyo.	The responses I produce without AI assistance seem less adequate or lower in quality than those I generate with its support.
11	Cuando leo textos, me cuesta trabajo identificar las ideas centrales sin que la IA me genere un resumen o "puntos clave".	When reading texts, I find it difficult to identify the main ideas unless AI provides me with a summary or “key points.”
12	Si la IA me da un resultado, me cuesta mucho editarlo o reescribirlo, prefiero usarlo casi tal cual lo generó.	When AI generates a result for me, I find it difficult to substantially edit or rewrite it and prefer to use it almost exactly as generated.

13	Si mi acceso a las herramientas de IA se interrumpe (e.g., por falta de internet), me siento inmediatamente ansioso/a o frustrado/a.	If my access to AI tools is interrupted (e.g., due to internet problems), I immediately feel anxious or frustrated.
14	Me pongo irritable o de mal humor si un profesor me pide completar una tarea en la que está prohibido usar cualquier tipo de IA.	I become irritable or frustrated when a professor asks me to complete an assignment in which any type of AI use is prohibited.
15	Cuando tengo una tarea difícil, mis pensamientos giran constantemente en torno a cómo voy a usar la IA para resolverla.	When I face a difficult academic task, my thoughts constantly revolve around how I will use AI to solve it.
16	He sentido un "vacío" o "bloqueo" mental al intentar estudiar o escribir sin tener la IA como soporte inmediato.	I have experienced a sense of “mental emptiness” or “blockage” when trying to study or write without AI as immediate support.
17	Para evitar la ansiedad, prefiero pagar o buscar una IA avanzada, aunque tenga que dedicarle un tiempo considerable a ello.	To avoid anxiety, I prefer to pay for or seek out more advanced AI tools, even if it requires considerable time or effort.
18	Si la IA me da un error o una respuesta irrelevante, siento un alto nivel de frustración que me dificulta seguir estudiando.	If AI provides an error or an irrelevant response, I experience a high level of frustration that makes it difficult for me to continue studying.
19	El tiempo que dedico a interactuar con la IA para la escuela me ha hecho descuidar otras responsabilidades o actividades de estudio importantes.	The time I spend interacting with AI for schoolwork has caused me to neglect other responsibilities or important study activities.
20	Debido a mi dependencia de la IA, a veces entrego trabajos que no reflejan mi nivel real de comprensión o conocimiento de la materia.	Due to my reliance on AI, I sometimes submit assignments that do not reflect my actual level of understanding or knowledge of the subject.
21	He tenido conflictos o he estado en riesgo de problemas éticos (plagio) debido a la manera en que he utilizado la IA en mis trabajos.	I have experienced conflicts or been at risk of ethical issues (e.g., plagiarism) because of the way I have used AI in my academic work.
22	Sé que al usar la IA estoy limitando el desarrollo de mis propias habilidades cognitivas (pensamiento crítico, síntesis), pero sigo usándola.	I am aware that using AI may limit the development of my own cognitive skills (e.g., critical thinking or synthesis), yet I continue to rely on it.
23	Mis interacciones con compañeros de clase sobre tareas han disminuido, pues prefiero obtener la solución directamente de la IA.	My interactions with classmates about academic tasks have decreased because I prefer obtaining solutions directly from AI.
24	Siento que si se me exigiera completar una materia sin absolutamente ninguna ayuda de IA, mi rendimiento se vería seriamente afectado.	I feel that if I were required to complete a course without any AI assistance at all, my academic performance would be seriously affected.

Supplementary Table 3. Spanish and English Wording of the Retained Items and Their Corresponding Factor Allocation

Spanish	English	Factor
Intento limitar el tiempo que utilizo la IA en mis estudios, pero siempre termino usándola más de lo que planeé.	I try to limit the amount of time I use AI for my studies, but I usually end up using it more than I planned.	Cognitive Reliance
Dejo de lado métodos de estudio más tradicionales (libros, apuntes) porque la IA me ofrece una solución más rápida y prioritaria.	I set aside more traditional study methods (e.g., books or class notes) because AI provides a faster and more immediate solution.	Cognitive Reliance
Siento que tengo que consultar a la IA inmediatamente al recibir una tarea académica, antes de intentar resolverla por mí mismo/a.	I feel the need to consult AI immediately when I receive an academic assignment, before trying to solve it on my own.	Cognitive Reliance
Si se me ocurre una idea para un ensayo o proyecto, mi primer impulso es pedirle a la IA que la desarrolle o la estructure.	When I come up with an idea for an essay or project, my first impulse is to ask AI to develop or structure it.	Cognitive Reliance
Me resulta difícil concentrarme en la lectura o el estudio si no tengo abierta una herramienta de IA lista para ser consultada.	I find it difficult to concentrate on reading or studying if I do not have an AI tool open and readily available.	Emotional-Dysregulated Reliance
Cada vez necesito usar la IA para tareas académicas más complejas (análisis crítico, síntesis, argumentación) que antes podía hacer solo/a.	I increasingly rely on AI for more complex academic tasks (e.g., critical analysis, synthesis, or argumentation) that I used to handle on my own.	Cognitive Reliance
Confío ciegamente en las respuestas y resúmenes de la IA, sin tomar el tiempo de verificar la información en fuentes primarias.	I trust AI-generated answers and summaries without taking the time to verify the information using primary sources.	Emotional-Dysregulated Reliance
Me siento mentalmente agotado/a si tengo que elaborar un texto argumentativo largo sin la asistencia directa de la IA.	I feel mentally exhausted when I have to write a long argumentative text without direct AI assistance.	Cognitive Reliance
Las respuestas que genero sin ayuda de la IA me parecen insuficientes o de menor calidad que las que obtengo con su apoyo.	The responses I produce without AI assistance seem less adequate or lower in quality than those I generate with its support.	Cognitive Reliance
Cuando leo textos, me cuesta trabajo identificar las ideas centrales sin que la IA me genere un resumen o "puntos clave".	When reading texts, I find it difficult to identify the main ideas unless AI provides me with a summary or “key points.”	Emotional-Dysregulated Reliance
Si la IA me da un resultado, me cuesta mucho editarlo o reescribirlo, prefiero usarlo casi tal cual lo generó.	When AI generates a result for me, I find it difficult to substantially edit or rewrite it and prefer to use it almost exactly as generated.	Emotional-Dysregulated Reliance

Si mi acceso a las herramientas de IA se interrumpe (e.g., por falta de internet), me siento inmediatamente ansioso/a o frustrado/a.	If my access to AI tools is interrupted (e.g., due to internet problems), I immediately feel anxious or frustrated.	Emotional-Dysregulated Reliance
Me pongo irritable o de mal humor si un profesor me pide completar una tarea en la que está prohibido usar cualquier tipo de IA.	I become irritable or frustrated when a professor asks me to complete an assignment in which any type of AI use is prohibited.	Emotional-Dysregulated Reliance
Cuando tengo una tarea difícil, mis pensamientos giran constantemente en torno a cómo voy a usar la IA para resolverla.	When I face a difficult academic task, my thoughts constantly revolve around how I will use AI to solve it.	Cognitive Reliance
He sentido un "vacío" o "bloqueo" mental al intentar estudiar o escribir sin tener la IA como soporte inmediato.	I have experienced a sense of “mental emptiness” or “blockage” when trying to study or write without AI as immediate support.	Emotional-Dysregulated Reliance
Para evitar la ansiedad, prefiero pagar o buscar una IA avanzada, aunque tenga que dedicarle un tiempo considerable a ello.	To avoid anxiety, I prefer to pay for or seek out more advanced AI tools, even if it requires considerable time or effort.	Emotional-Dysregulated Reliance
Si la IA me da un error o una respuesta irrelevante, siento un alto nivel de frustración que me dificulta seguir estudiando.	If AI provides an error or an irrelevant response, I experience a high level of frustration that makes it difficult for me to continue studying.	Emotional-Dysregulated Reliance
El tiempo que dedico a interactuar con la IA para la escuela me ha hecho descuidar otras responsabilidades o actividades de estudio importantes.	The time I spend interacting with AI for schoolwork has caused me to neglect other responsibilities or important study activities.	Emotional-Dysregulated Reliance
Debido a mi dependencia de la IA, a veces entrego trabajos que no reflejan mi nivel real de comprensión o conocimiento de la materia.	Due to my reliance on AI, I sometimes submit assignments that do not reflect my actual level of understanding or knowledge of the subject.	Emotional-Dysregulated Reliance
Sé que al usar la IA estoy limitando el desarrollo de mis propias habilidades cognitivas (pensamiento crítico, síntesis), pero sigo usándola.	I am aware that using AI may limit the development of my own cognitive skills (e.g., critical thinking or synthesis), yet I continue to rely on it.	Cognitive Reliance
Mis interacciones con compañeros de clase sobre tareas han disminuido, pues prefiero obtener la solución directamente de la IA.	My interactions with classmates about academic tasks have decreased because I prefer obtaining solutions directly from AI.	Emotional-Dysregulated Reliance
Siento que si se me exigiera completar una materia sin absolutamente ninguna ayuda de IA, mi rendimiento se vería seriamente afectado.	I feel that if I were required to complete a course without any AI assistance at all, my academic performance would be seriously affected.	Emotional-Dysregulated Reliance
